# Influence of Water on the Structure and Dielectric Properties of the Microcrystalline and Nano-Cellulose

**DOI:** 10.1186/s11671-017-2231-5

**Published:** 2017-07-26

**Authors:** Kostiantyn M. Kovalov, Olexander M. Alekseev, Maxim M. Lazarenko, Yu F. Zabashta, Yurii E. Grabovskii, Sergii Yu Tkachov

**Affiliations:** Taras Shevchenko National University of Kyiv, Volodymyrska str. 64/13, Kyiv, 01601 Ukraine

**Keywords:** Microcrystalline cellulose, Relaxation, X-ray, Dielectric permittivity

## Abstract

Influence of water in the different states on a structure and dielectric properties of microcrystalline cellulose were studied by of X-ray, thermogravimetry, and dielectric spectroscopy. At research of microcrystalline cellulose (MCC) with different content of water, it is shown that the molecules of water are located in the macropores of MCC and in multimolecular hydrated layers. It is shown that at the increase of concentration of water in a hydrated shell, the reorganization of molecules of cellulose in the surface of crystallites takes place, and as a result, their transversal size and crystallinity increase. It is shown that during the concentration of water, more than 13% in a continuous hydrated shell of crystallites appears. Temperature dependences of actual and imaginary parts of complex dielectric permittivity were studied in the interval of temperatures [−180 ÷ 120] °C on frequencies of *f* = 5, 10, 20, and 50 kHz. A low-temperature relaxation process and high-temperature transition were observed. Low-temperature relaxation process which is related to transition of surface methylol groups of molecules of cellulose conformation from *tg* to *tt* is shifted toward low temperatures at the increase of concentration of water in microcrystalline cellulose.

## Background

Usual cellulose from various vegetative sources, which is practically inexhaustible renewable ecologically clean resource, is a raw material for production of microcrystalline cellulose (MCC). It determines the growing interest for investigation of its physical and physicochemical properties. Another important factor, which attracts attention of investigators, is availability of the crystalline particles in the MCC structure, the study of properties of which is promising for many directions of development of modern technologies. The most developed from such directions are pharmaceutical industry and cosmetics [1]; however, currently, MCC begins to be used as a filler in composite materials [2], modern electronics [3], and laser optics [4, 5]. In this regard, the ability of MCC to absorb moisture, which can substantially affect on its properties, in particular, structural [6], electrical [7, 8], and thermophysical [9, 10] ones, is important.

## Methods

### The Samples

The samples of MCC (Cellets-100) grade, produced by Shin-Etsu Company (Japan), were used for investigation. Initial MCC was dispersed in an agate mortar. To obtain water-free samples, the samples were held in the drying box during 3 days at the temperature of 115 °C, and then, they were encapsulated in the vacuum press mold. The samples with different level of moisture were obtained by their holding during different times in under saturated water vapor.

### Equipment

Analysis of the sample structure was performed using X-ray diffractometer DRON-3M with the tube BSV-28 (*λ* = 1.54178 Å).

Differential thermal analysis (DTA) and thermogravimetric investigations were performed using derivatograph Q-1500D. Investigations were carried out within the temperature range *T* = 20 ÷ 250° with the rate of 5 °C/min.

The samples for dielectric investigations were produced by the compacting of MCC powder between coatings from a stainless steel at the pressure of 120 kg/cm^2^. Then, the sample with laminated coatings was placed in the thermostabilized four-electrode cell, which permitted to control the sample thickness during measurements using an additional air-dielectric capacitor. Measurements of the capacity and loss factor of this cell on four different frequencies *f* = 5, 10, 20, 50 kHz within the temperature range (−180 ÷ 120) °C were performed using automated installation based on the alternating current bridge P5083 [11].

## Results and Discussion

### Thermogravimetric Investigations

Investigations of the amount of water in MCC were performed using derivatograph Q-1500D. In the investigated sample, the temperature (*T*), mass change (*m*), and rate of the mass change (*dm/dT*) were measured simultaneously and differential and thermal analyses (DTA) were performed.

**Fig. 1:**
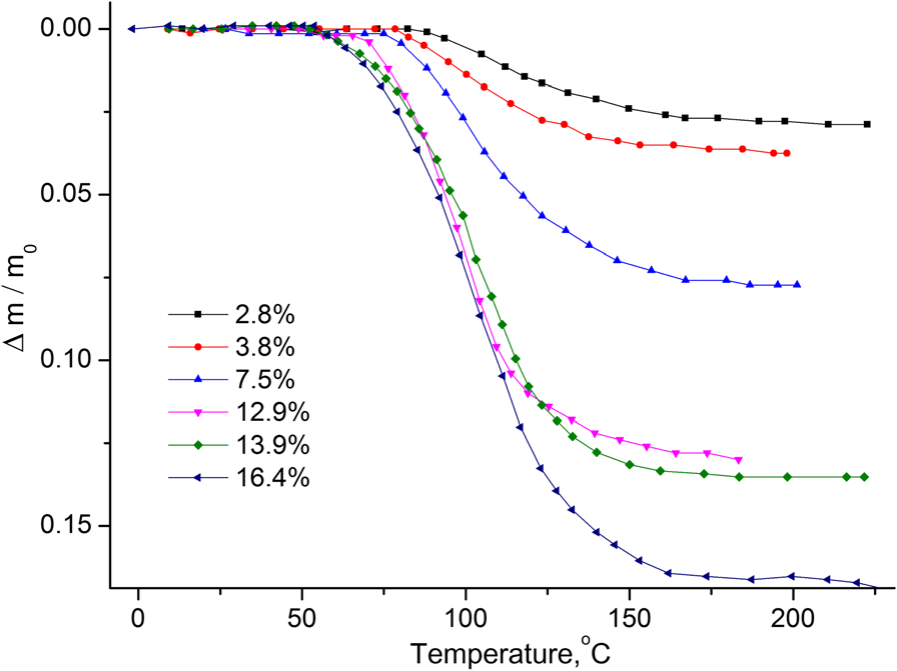
Temperature dependence of the relative mass change of MCC. Temperature dependence of the relative mass change Δ*m*/*m*_0_ of MCC samples with different water content

**Fig. 2:**
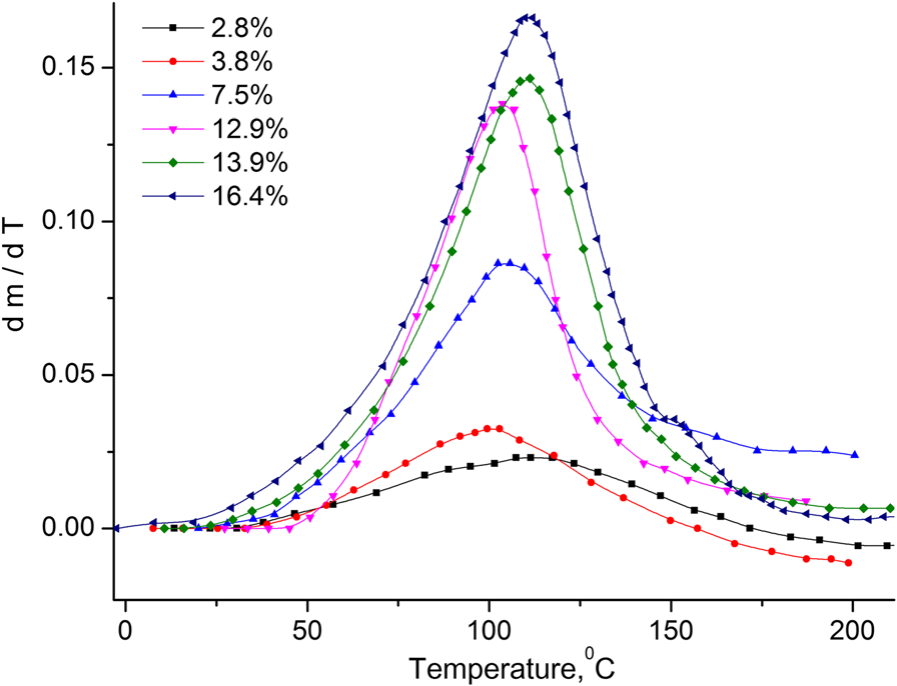
Temperature dependence of the derivative of mass change of MCC. Temperature dependence of the derivative of mass change *dm*/*dT* of MCC samples with different water content

Temperature dependencies of the relative mass change *Δm*/*m*_0_ *=* (*m−m*_0_)/*m*_*0*_, *m*_*0*_––initial sample mass (see Fig. 1) and derivative of the mass change *dm/dT* (see Fig. 2) were obtained.

**Fig. 3:**
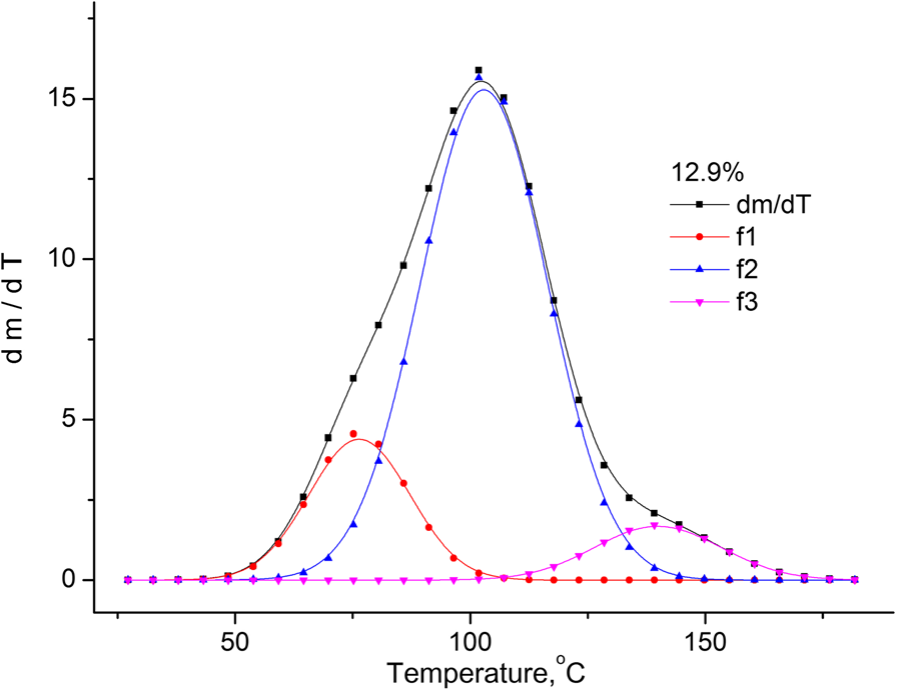
Approximation of the *dm*/*dT* dependence for MCC sample with the humidity of 12.9%. Approximation of the *dm*/*dT* dependence for MCC sample with the humidity of 12.9% using Gauss distribution with three peaks

One can consider that the loss of mass by the sample is caused by the evaporation of water, which is located in the sample in different states [12, 13]. Therefore, the *dm*/*dT* was divided on peaks using the Gauss distribution (see Fig. 3).

The dependence of *dm*/*dT* for each of the investigated samples can be described by the superposition of three peaks. The first peak (*f*_1_) is connected with the evaporation of water of physical-mechanical bond with MCC, which is located in the micropores of the sample, the second one (*f*_2_) is caused by the water of polymolecular and monomolecular hydrate layers, and the third peak is caused by the thermal oxidative degradation of the sample.

In Table 1, percentage *S*_1_ in the square of the general peak *dm*/*dT* and mass percentage *w*_*i*_ = *CS*_*i*_/100% into general mass of different types of water and destruction for the MCC samples with different water content is shown*.*

**Table 1:** Contributions of different types of water and destruction for the MCC samples with different water content in the square of the general peak on the graph dm/dT and its content in the samples

C (general water content in MCC), %	I (*f*_1_)	I (*f*_1_)	II (*f*_2_)	II (*f*_2_)	III (*f*_3_)	III (*f*_3_)
	*S*_1_, %	*W*_1_, %	*S*_2_, %	*W*_2_, %	*S*_3_, %	*W*_3_, %
2.8	–	–	100	2.8	–	–
3.8	3.3	0.1	88.7	3.4	7.9	0.3
7.5	7.1	0.5	84.3	6	8.5	0.6
12.9	15	1.9	77	10	7.5	1
13.9	21	3.1	72	10	6	0.8
16.4	26	4	67	10.7	6.8	1

It is seen that the amount of physically mechanically bound water *S*_1_ increases with the growth of the content of general water, and the amount of water in hydrate shells *S*_2_ also increases and reaches its saturation at concentration of the general water of 12.9%.

Let us consider in more details the second peak (*f*_2_), which is caused by cellulose crystals dehydration.

The method for calculation of kinetic parameters of the dehydration process, such as the energy of activation *E* and pre-exponential factor (*k*_0_) are described in [12]. Kinetic equation of the desorption process may be presented as:

1 $$ \frac{dQ}{dt}=-k{Q}^n,k=\frac{-dQ/dt}{Q^n}. $$

A degree of covering of cellulose crystallites by the water molecules (*Q*) changes from 1 (for initial material) to 0 (whole water is dehydrated). An order of reaction (*n*) is an integer from 1 to 3; it is assumed that it is known from the experiment. The constant of the reaction rate (*k*) can be written as:

2 $$ k={k}_0 \exp \left(-\frac{E}{RT}\right), $$

where *R* is universal gas constant. The value of *E* is taken in this approximation as a constant, which means an equivalence of all hydration centers of the MCC crystals surface. The pre-exponential factor can be written as *k*_0_ *= ZP*, where *Z* is the theoretical number of discontinuities of hydrogen bonds between water molecules and cellulose (MCC) in a hydrate layer per unit time, and *P* is a probability factor that takes into account all the effects caused by a deviation from ideality. The probability of breaking the hydrogen bond between the water molecule and the cellulose is much greater at a higher concentration of water molecules in the hydrated shell, so the water molecule after the bond break does not form a new bond with the neighboring free node, but translates from the hydrate layer. Therefore, the pre-exponential factor of the reaction rate is small at the low concentrations of water and increases with the growth of water concentration in the hydrated shell. When saturation of the hydrated shell is achieved (all nodes are occupied), then *k*_0_ does not depend on the concentration of the moisture. This is observed for the MCC water system at a concentration more than 12.9%.

After substitution of Eq. 1 in Eq. 2 and logarithmation, we obtain:

3 $$ \ln k= \ln \left[\frac{-dQ/dt}{Q^n}\right]= \ln {k}_0-\frac{E}{RT}. $$

Under initial conditions *Q*_*t=*0_ *=* 1, *Q*_*t=∞*_ *=* 0 and constant heating rate (*β*), i.e., at linear dependence of the temperature on time

4 $$ T(t)={T}_0+\beta t, $$

the following relationships are fulfilled

5 $$ Q(t)=\frac{S_T}{S_0};-\frac{dQ}{dt}=\beta \frac{f_3}{S_0}, $$

where *S*_*0*_ and *S*_*T*_ are the areas on the plot *f*_*2*_ under the whole peak and the part of the peak from *T* to ∞

6 $$ {S}_T={\displaystyle \underset{T}{\overset{\infty }{\int }}{f}_2dT,{S}_0=}{\displaystyle \underset{0}{\overset{\infty }{\int }}{f}_2dT}. $$

If all assumptions, which are placed in this method are correct and the order of reaction *n* is chosen correctly, then the dependence $\ln \left[\frac{-dQ/dt}{Q^n}\right]$ on the inverse temperature (Eq. 3) is linear in the whole temperature range. Having experimental values of *f*_*3*_ and *β*, using expressions Eqs. 5 and 6, one can obtain *Q* and *dQ*/*dt*, and parameters of non-isothermal kinetics *k*_0_ and *E* are calculated from Eq. 3.

An advantage of this method is the usage of the whole array of experimental data, including high temperature part of the thermogram, which is especially important at determination of the order *n*, determination of the reaction mechanism, and model fairness.

**Fig. 4:**
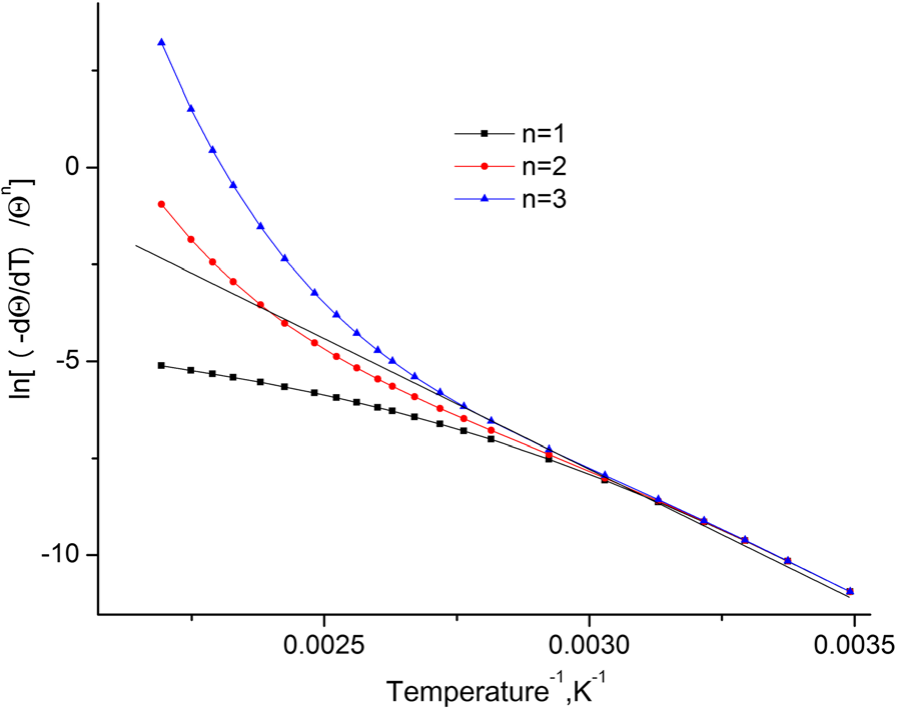
Dependence of $\ln \left[\frac{-dQ/dt}{Q^n}\right]$ on the reverse temperature for different orders of reaction. Dependence of $\ln \left[\frac{-dQ/dt}{Q^n}\right]$ on the reverse temperature for different orders of reaction

The dependences of $\ln \left[\frac{-dQ/dt}{Q^n}\right]$ from a reverse temperature for the investigated standards (Fig. 4) were built. These dependences are approximated by straight lines by means of least-squares method. It is found out that the highest coefficient of Pearson is observed at *n* = 2.

**Fig. 5:**
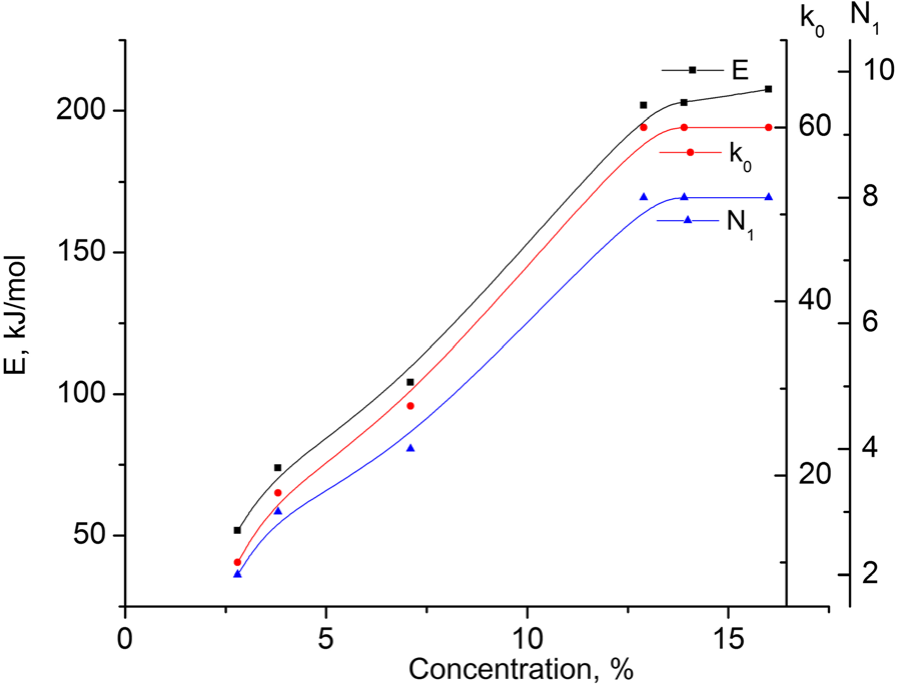
Dependencies of activation energy (*E*), pre-exponential factor (*k*_*0*_), and number of hydrogen bonds *N*_*1*_ for the samples of MCC. Dependencies of activation energy (*E*), pre-exponential factor (*k*_*0*_), and number of hydrogen bonds *N*_*1*_ for the samples of MCC with different water content

Calculated activation energy (*E*) and pre-exponential factor (*k*_*0*_) of the investigated MCC samples are presented in Table 2 (see Fig. 5). Assuming that the activation energy is connected with the rapture of the hydrogen bonds, which comprise 25 *k*J/mol [10], it is possible to calculate their number *N*_*1*_ (see Table 2).

**Table 2:** Dependencies of activation energy (*E*), pre-exponential factor (*k*_*0*_), and number of hydrogen bonds *N*_*1*_ on water content in the investigated samples

C (water content in MCC), %	*k*_*0*_	E, kJ/mol	*N*_1_
2.8	10	51.75	2
3.8	18	73.92	3
7.1	28	104.19	4
12.9	60	201.91	8
13.9	60	202.82	8
16.4	60	207.58	8

It is seen that with the growth of concentration of physically and chemically bound water, the increase of activation energy (*E*) is observed, and at concentration of 12.9%, it reaches its saturation. The value of activation energy for the saturated state corresponds to eight hydrogen bonds.

Therefore, we can assume that at water concentration of 12.9%, the continuous hydrate shell of MCC crystals is formed in the sample.

### X-ray Diffraction Analysis

**Fig. 6:**
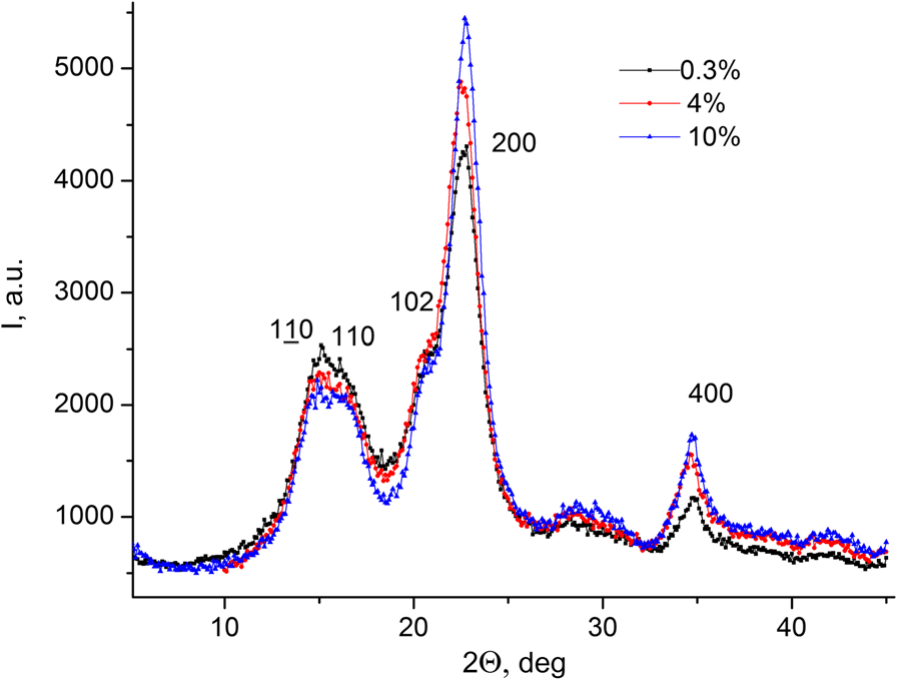
Dependencies of intensity of diffracted X-ray radiation for MCC. Dependencies of intensity of diffracted X-ray radiation for MCC with different water concentrations on the diffraction angle *I*(2*θ*)

The dependencies of diffracted X-ray radiation intensity for microcrystal cellulose with different water concentrations on diffraction angle *I*(2*θ*) within the angles range [5 ÷ 45]°(see Fig. 6) with the step of angle change 0.1° were obtained.

Maxima, which correspond to different crystallographic plains, are observed on the MCC X-ray diffractogram (see Fig. 6).

The degree of crystallinity (*C*_*k*_) of MCC was calculated by the integral intensity of X-rays scattering as a ratio of structural reflexes to total intensity of the diffraction pattern excluding background in the range 2*θ* [5 ÷ 45]° (see Fig. 6). Data of *C*_*k*_ for MCC samples are presented in Table 3 [14].

**Table 3:** Transversal dimensions of crystallites *B*_hlk_ and degree of crystallinity *C*_*k*_ for the samples of MCC with different content of water

C (water content in MCC), %	0.3	4	10
*B*_110_, nm	4.21	4.35	4.62
*B*_110_, nm	4.50	4.68	4.91
*B*_102_, nm	5.98	6.14	6.37
*B*_200_, nm	4.58	4.73	4.98
*B*_004_, nm	6.1	6.57	7.03
*C*_*к*_, %	58	65	68

Transverse dimensions of cellulose crystallites in normal direction to the system were calculated by Debye–Sherrer formula [15].

7 $$ {B}_{\mathrm{hkl}}=\frac{k\lambda }{h\cdot \cos {\theta}_{\mathrm{hkl}}}, $$

where *k* = 0.94---dimensionless form-factor, λ---wavelength of CuKα-radiation (1.54178 Å); *h*---half-width of (hlk) reflex, *θ*_hlk_---angle of diffraction from the (hlk) plains system. Urotropine was used as an etalon for higher confidence. Instrumental error was estimated using the etalon, and corrections for the reflex half-width were calculated.

Data of dimensions *B*_hlk_ for MCC samples with different water content and crystallinity are presented in Table 3.

**Fig. 7:**
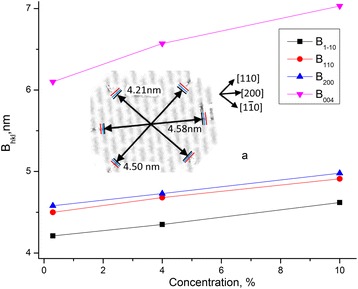
Dependencies of the dimensions of crystallite of MCC *B*_hlk_ on water content. Dependencies of the dimensions of crystallite of MCC *B*_hlk_ on water content, a---transversal dimensions of MCC crystallite (*black*) and growth of its dimension with the humidity increase (*blue*---MCC + 4% and *red*---MCC + 10%)

It follows from the obtained results (Table 3 and Fig. 7) that transversal dimension of crystallites and crystallinity grow with water concentration. Growth of dimensions takes place by different directions on approximately 0.4 nm. Average diameter of cellulose molecule is of an order of *d* = 0.8 nm. Therefore, with the growth of water concentration, an ordering of cellulose molecules in the boundary layer may happen, and, as a consequence, increase of transversal dimension of cellulose crystallites and crystallinity can take place.

### Dielectric Properties of MCC

**Fig. 8:**
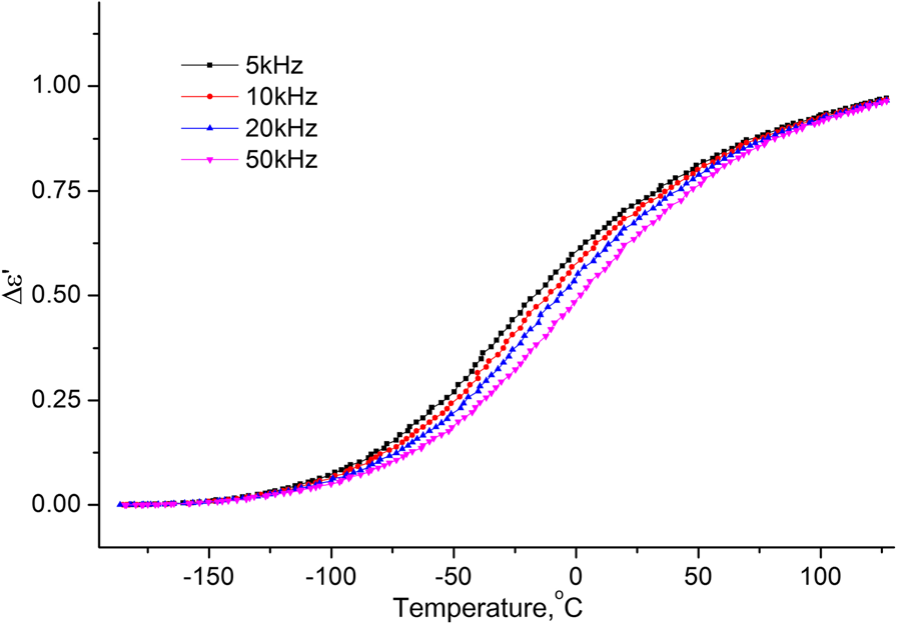
Temperature dependence of the change of increment of real part of the complex dielectric permittivity. Temperature dependence of the change of increment of real part (Δ*ε*′ = *ε*′(*T*) − *ε*_∞_, ${\varepsilon}_{\infty }={\left.{\varepsilon}^{\prime}\right|}_{T=-180{}^o\mathrm{C}}$) of the complex dielectric permittivity MCC + 0.3% H_2_O on the frequencies 5, 10, 20, and 50 kHz

**Fig. 9:**
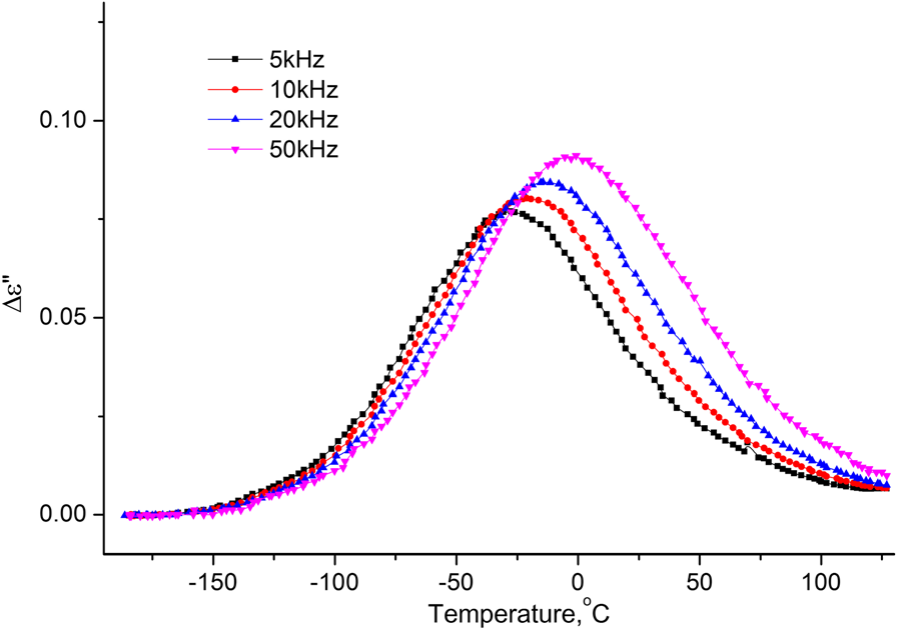
Temperature dependence of imaginary part of the dielectric permittivity of MCC + 0.3% H_2_O. Temperature dependence of imaginary part of the dielectric permittivity of MCC + 0.3% H_2_O on frequencies 5, 10, 20, and 50 kHz

Temperature dependencies of the real and imaginary parts of the complex dielectric permittivity within the temperature range [−180 ÷ 120] °C on the frequencies *f* = 5, 10, 20, and 50 kHz for the samples MCC + 0.3% H_2_O (see Figs. 8 and 9) were investigated.

From the two processes observed on the temperature dependences of ∆*ε*′(*T*) and *ε*′′(*T*), the low temperature is a relaxation since the maximum on ∆*ε*′(*T*) and the inflection on *ε*′′(*T*) is shifted with the increasing of the frequency. Usually, this process is called the β-process, its molecular mechanism is not finally established. We assume that it is related to the reorientation of methylol groups on the surface of the crystallites of the MCC, and this reorientation occurs with a change in the conformation of the methylol groups *tg* to *tt*, the breakup of the intra-molecular H bond and the formation of the H bond with the adsorbed water molecule [16].

**Fig. 10:**
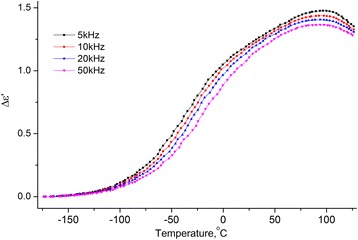
Temperature dependence of the increment of real part of the dielectric permittivity MCC + 2.8% H_2_O. Temperature dependence of the increment of real part (Δ*ε*′ = *ε*′(*T*) − *ε*_∞_, where ${\varepsilon}_{\infty }={\left.{\varepsilon}^{\prime}\right|}_{T=-180{}^oC}$) of the dielectric permittivity MCC + 2.8% H_2_O on frequencies 5, 10, 20, and 50 kHz

**Fig. 11:**
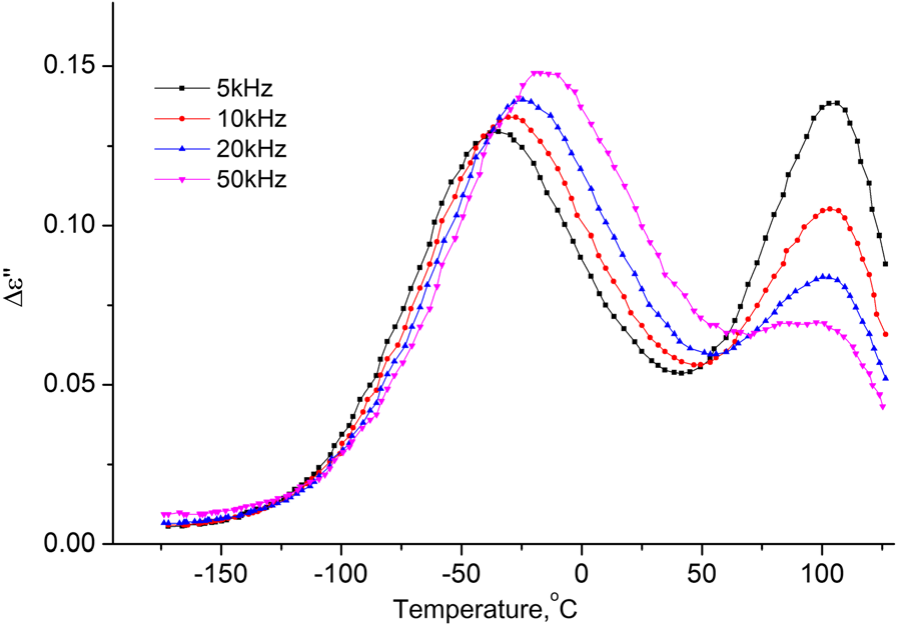
Temperature dependence of imaginary part of the dielectric permittivity of MCC + 2.8% H_2_O. Temperature dependence of imaginary part of the dielectric permittivity of MCC + 2.8% H_2_O on frequencies 5, 10, 20, and 50 kHz

The high-temperature process on the dependences ∆*ε*′(*T*)and *ε*′′(*T*) does not shift with the frequency variation, its intensity grows with the concentration of the water in the MCC (see Figs. 10 and 11). We assume that the reason for this is the desorption of the water with increasing temperature with its subsequent condensation in pores, which leads to a marked increase in the temperature *ε*′ and *ε*′ of the sample. With the further increase in temperature, the evaporation of water occurs, and ∆*ε*′(*T*) and *ε*′′(*T*) decrease.

**Fig. 12:**
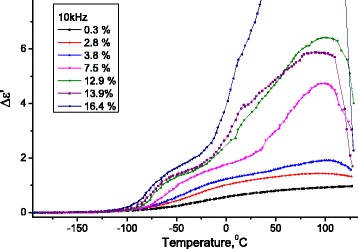
Temperature dependence of the change of increment of the real part of the dielectric permittivity of MCC. Temperature dependence of the change of increment of the real part (Δ*ε*′ = *ε*′(*T*) − *ε*_∞_, where ${\varepsilon}_{\infty }={\left.{\varepsilon}^{\prime}\right|}_{T=-180{}^oC}$) of the dielectric permittivity of MCC samples with different water content

In Fig. 12, dependences of the increment of real part (*Δε*′ = *ε*′(*T*) − *ε*_∞_, where ${\varepsilon}_{\infty }={\left.{\varepsilon}^{\prime}\right|}_{T=-180{}^oC}$) of the dielectric permittivity on temperature on frequency 10 kHz are presented for investigated samples of MCC with different water contents.

It was shown in [16] that low-temperature relaxation process, which is connected with transition of methylol group from *tg* to *tt* conformation, can be described by the model with one relaxation time [17]

8 $$ \tau =\frac{2\pi }{\omega_0}\frac{ \exp \left(\raisebox{1ex}{$U$}\!\left/ \!\raisebox{-1ex}{$kT$}\right.\right)}{1+ \exp \left(\raisebox{1ex}{$-V$}\!\left/ \!\raisebox{-1ex}{$kT$}\right.\right)} $$

and for the increment of dielectric permittivity the following formula is valid:

9 $$ \varDelta \varepsilon ={\varepsilon}^{\prime }(T)-{\varepsilon}_{\infty }=\frac{N{\mu}^2}{3k{\varepsilon}_0T}\cdot \frac{ \exp \left(\raisebox{1ex}{$-V$}\!\left/ \!\raisebox{-1ex}{$kT$}\right.\right)}{{\left[1+ \exp \left(\raisebox{1ex}{$-V$}\!\left/ \!\raisebox{-1ex}{$kT$}\right.\right)\right]}^2}, $$

where *ε*′*(T)––*is the dielectric permittivity of the sample at temperature *T*, *N* is the concentration of relaxation oscillators, *μ* ^2^ is the average square of difference of dipole moments of relaxation oscillators in two positions of equilibrium, and *V* is the difference of energies of relaxation oscillators in these equilibrium positions.

During approximation of the dependence of increment of real part of complex dielectric permittivity for MCC samples with different humidity by the dependence (Eq. 9), values of *N* and *V* were obtained (see Table 4) with regard for difference of dipole moments of relaxation oscillators in two equilibrium positions, which was calculated with regard for space structure of methylol group *μ* = 5.57*D* = 18.381 ⋅ 10^− 30^ *C* ⋅ *m*.

**Table 4:** Concentration of relaxation oscillators *N* and energies difference *V* for the samples of MCC with different water content

C (water content in MCC), %	0.3	2.8	3.8	7.5	12.9	13.9	16.4
*V*, *k*J/mole	6.7	6.7	6.7	6.7	7	7	7.3
*N*, 10^23^ m^−3^	40	65	76	117	139	150	213

It is seen from Table 4 that at the growth of water content, energy difference *V* does not change, but concentration of methylol groups, which contributes in dielectric relaxation, increases.

**Fig. 13:**
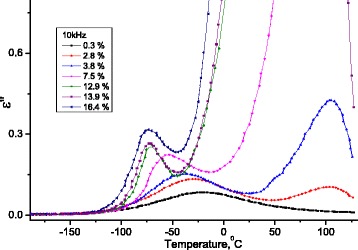
Temperature dependence of imaginary part of dielectric permittivity for MCC samples with different water content. Temperature dependence of imaginary part of dielectric permittivity for MCC samples with different water content

In Fig. 13, dependencies of imaginary part of dielectric permittivity on temperature on the frequency *f* = 10 kHz for studied samples of MCC with different water content are presented.

**Fig. 14:**
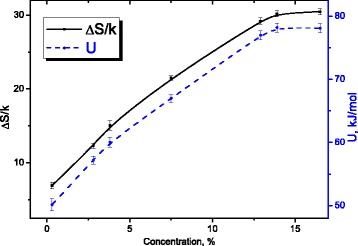
Dependencies of the entropy and the activation energy of relaxation process on water concentration in MCC. Dependencies of the entropy and the activation energy of relaxation process on water concentration in MCC

For relaxation process under approximation of one time of relaxation (*τ* = *τ*_0_ exp((*U* − *TΔS*)/*kT*) and under condition of maximum of dependence of the imaginary part of complex dielectric permittivity (*ε* ′ ′ (*T*, *f*)) *ωτ* = 1_,_ changes of entropy *ΔS*/*k* (see Fig. 14) and activation energy *U* (see Fig. 14) with water concentration were obtained.

We assume that entropy of activation of the relaxation process is connected with the probability of formation of hydrogen bonds of methylol group with surrounding molecules of water [18].

Its growth testifies to the increase of the average number of water molecules, which surround these groups on the surface of MCC crystallites.

The saturation of the dependence ∆*S/k* (see Fig. 14) at growth of the water concentration is a consequence of formation of solid hydrate shell in the system of MCC crystallites as a result of incorporation of separate surface clusters from water molecules after reaching of their threshold concentration, which is determined by available water specific surface of MCC crystallites system during their swelling.

The growth of activation energy *U*(*C*) of the relaxation process (see Fig. 14) and saturation of its dependence on water concentration has the same nature as for entropy activation is indicative of structuring of the surface of MCC crystallites during formation of their solid hydrate shell.

## Conclusions

Studies of dielectric, thermophysical, and structural properties of MCC samples with different degree of humidity were performed. The existence of relaxation process, which is connected with reorientation of the surface methylol groups of cellulose molecule by the change of their conformations from *tg* to *tt* is shown.

During the holding of the MCC sample in the saturated water vapor, the hydrate shell begins to form gradually on the surface of cellulose crystallites. It structures the boundary layer of MCC crystallites. At water concentration, higher than 13% formation of solid hydrate shell on MCC crystallites is observed.

Formation of hydrate shell results in the shift of relaxation process to the side of low temperatures due to its influence on the potential barrier and change of vibrations in equilibrium positions of surface methylol groups of the cellulose molecule at their transition from conformation *tg* to *tt*.
